# Evaluation of Intraoperative Gentamicin Bladder Irrigation for Mitigation of Urinary Tract Infections After Kidney Transplantation: A Propensity Score–Matched Analysis of a Randomized Controlled Trial

**DOI:** 10.1155/joot/9377493

**Published:** 2025-11-29

**Authors:** Aviad Gravetz, Vladimir Tennak, Mais Khamis, Vadym Mezhybovsky, Michael Gurevich, Sigal Eisner, Tasnim Lubani, Dana Bielopolski, Alaa Atamna, Fahim Kanani, Eviatar Nesher

**Affiliations:** ^1^Department of Transplantation, Rabin Medical Centre, Petah Tikva, Israel; ^2^Gray Faculty of Medical and Health Sciences, Tel Aviv, Israel; ^3^Department of Nephrology, Rabin Medical Centre, Petah Tikva, Israel; ^4^Department of Infectious Diseases, Rabin Medical Centre, Petah Tikva, Israel

**Keywords:** bladder irrigation, gentamicin, infection prevention, kidney transplantation, propensity score matching, sex differences, urinary tract infection

## Abstract

**Background:**

Urinary tract infections (UTIs) remain the leading infectious complication after kidney transplantation. We evaluated intraoperative gentamicin bladder irrigation as a novel preventive strategy.

**Methods:**

In this randomized, double-blind controlled trial at a tertiary transplant center (January-December 2021), 147 kidney transplant recipients were randomized to receive intraoperative bladder irrigation with gentamicin (160 mg/250 mL) or saline during ureteroneocystostomy. Due to baseline imbalances, propensity score matching yielded 49 matched pairs. The primary endpoint was UTI incidence within 30 days post-transplant, defined by both microbiological (≥ 10^5^ CFU/mL) and clinical criteria.

**Results:**

UTI incidence was 26.5% (13/49) in controls versus 16.3% (8/49) with gentamicin (absolute risk reduction 10.2%, *p*=0.325). The number needed to treat was 10 overall. Striking sex-specific differences emerged: females demonstrated 40.7% baseline UTI risk versus 14.1% in males (*p*=0.004). Gentamicin efficacy varied markedly by sex, with the NNT of 5 for females (50.0%–30.8%) versus 17 for males (17.1%–11.1%). Living donor recipients showed greater benefit (NNT = 7) than deceased donor recipients (NNT = 23). No adverse events were attributable to gentamicin, with similar rates of bacteremia and surgical site infections between groups.

**Conclusions:**

Intraoperative gentamicin bladder irrigation safely reduced early post-transplant UTIs by 38.5%, with efficacy in female recipients. While underpowered for statistical significance, the clinically meaningful effect size and excellent safety profile support considering this intervention for high-risk recipients, especially females, pending larger confirmatory trials.

## 1. Introduction

Despite advances in transplant medicine, urinary tract infections (UTIs) remain the most common infectious complication after kidney transplantation, affecting 34%–47% of recipients within the first year [1–7]. Early UTIs, particularly within 30 days post-transplant, coincide with maximal immunosuppression and are associated with increased risk of acute rejection, graft dysfunction, and healthcare utilization [8–11]. The emergence of multidrug-resistant organisms further complicates management in this vulnerable population [12–14].

Current preventive strategies, including systemic antibiotic prophylaxis and early device removal, have shown variable efficacy with no consensus on optimal approaches [15–18]. Intravesical antimicrobial instillation offers an alternative strategy that achieves high local drug concentrations while minimizing systemic exposure. Gentamicin bladder irrigation has demonstrated efficacy in preventing UTIs in catheterized patients and those with neurogenic bladder [19, 20]. While Salehipour et al. reported promising results with perioperative bladder irrigation in transplant recipients, this approach remains unvalidated [21].

Internal audit data from our transplant program revealed that nearly half of kidney recipients developed UTIs, with approximately one in 10 progressing to urosepsis. This substantial burden, combined with the lack of consensus on optimal preventive strategies and absence of robust evidence for current approaches, prompted us to investigate the irrigation intervention. We sought to evaluate the safety and efficacy of intraoperative gentamicin bladder irrigation in reducing post-transplant UTIs and to identify which patient subgroups might derive the greatest benefit. This randomized controlled trial tested whether gentamicin irrigation, administered during routine bladder filling for ureteroneocystostomy, could safely reduce early UTI incidence across different recipient populations.

## 2. Methods

### 2.1. Study Design and Setting

This prospective, randomized, double-blind, controlled trial was conducted at Rabin Medical Center, a tertiary transplant center in Israel, between January and December 2021. The study protocol received institutional review board approval (IRB 0254-23-RMC) and was designed in accordance with CONSORT guidelines for reporting randomized trials. While a comprehensive protocol was established prior to study initiation, technical difficulties prevented trial registration. Nevertheless, we maintained rigorous adherence to the predetermined protocol throughout the study period. All participants provided written informed consent before enrollment, and the study was conducted according to Declaration of Helsinki principles.

### 2.2. Study Population

We enrolled consecutive adult kidney transplant recipients at our center between January and December 2021. Eligible participants included all adults (≥ 18 years) receiving kidney transplants from either living or deceased donors during the study period. Both first-time and repeat transplant recipients were included to enhance generalizability.

Patients were excluded if they had known allergies to aminoglycosides, active UTIs at transplantation (defined by positive urine culture or clinical symptoms), or had received systemic antibiotics within 7 days before surgery (standard perioperative prophylaxis excepted). Additional exclusion criteria included anatomical urinary diversions, multiorgan transplant recipients, pregnancy or lactation, concurrent enrollment in other interventional trials, or inability to provide informed consent. Throughout the enrollment period, we maintained detailed records of all screened patients to ensure transparent reporting according to CONSORT guidelines.

### 2.3. Randomization and Blinding

Participants were randomized 1:1 to receive either gentamicin bladder irrigation or saline control using computer-generated block randomization with variable block sizes [4, 6, 8]. Randomization was stratified by induction therapy type (basiliximab vs. antithymocyte globulin).

To ensure double-blinding, the hospital pharmacy prepared identical-appearing irrigation solutions labeled only with study numbers. Both solutions were prepared fresh for each case: one containing 160-mg gentamicin in 250-mL normal saline, and one containing normal saline alone. The surgical team, nursing staff, patients, and outcome assessors remained blinded to treatment allocation throughout the study period. Only the preparing pharmacist had access to the randomization list.

### 2.4. Intervention

#### 2.4.1. Bladder Irrigation Protocol

The intervention was integrated into the standard surgical technique during kidney transplantation:

1. Preparation Phase: Prior to transplantation, a sterile irrigation set was assembled under aseptic conditions. This included:
  ◦ A three-way Foley catheter inserted after anesthesia induction
  ◦ Connection of one port to the irrigation solution bag (positioned on an elevated IV pole)
  ◦ Connection of the drainage port to a collection bag
  ◦ Maintenance of closed system integrity
2. Preirrigation Sampling: The first urine specimen was collected sterilely for baseline culture before irrigation commenced.
3. Irrigation Timing: Upon completion of vascular anastomoses and graft reperfusion, as the surgeon prepared for ureteroneocystostomy, the nurse initiated bladder irrigation by gravity flow. This timing was critical as:
  ◦ Many recipients had minimal pretransplant urine output resulting in a collapsed bladder
  ◦ Bladder distension facilitated identification and safe cystotomy creation
  ◦ The antimicrobial solution (if randomized to gentamicin) had maximal contact time during ureteral implantation
4. Irrigation Technique:
  ◦ Solution flowed by gravity until moderate bladder distension achieved (typically 200–250 mL)
  ◦ The irrigation was clamped during ureteroneocystostomy
  ◦ After ureteral stent placement and cystotomy closure, the bladder was emptied
  ◦ No additional irrigation was performed postoperatively

### 2.5. Control Group

Patients randomized to control received identical bladder irrigation with normal saline alone, following the same protocol.

### 2.6. Study Endpoints and Surveillance

Our primary endpoint was the incidence of UTI within 30 days post-transplant. We adopted stringent diagnostic criteria modified from KDIGO and AST guidelines [22, 23], requiring both microbiological and clinical evidence for diagnosis. Microbiologically, UTI was defined as a positive urine culture yielding ≥ 10^5^ colony-forming units per milliliter of a single uropathogen, or ≥ 10^4^ CFU/mL when accompanied by clinical symptoms. Clinical criteria included either lower tract symptoms (dysuria, urgency, frequency, or suprapubic pain) or upper tract involvement manifested by fever above 38°C, graft tenderness, elevated inflammatory markers, or bacteremia with the same organism.

Secondary endpoints encompassed time to first UTI, incidence of bacteremia and surgical site infections, graft function assessed by serum creatinine at discharge and 30 days, length of hospitalization, 30-day mortality, and any adverse events potentially attributable to gentamicin exposure.

We implemented systematic microbiological surveillance with urine cultures obtained at baseline (preirrigation), and on postoperative days 3 and 5, supplemented by additional cultures whenever clinical suspicion arose. This intensive monitoring protocol aimed to capture both symptomatic infections and asymptomatic bacteriuria, though only patients meeting both microbiological and clinical criteria received treatment. Antibiotic therapy, when indicated, was tailored according to culture sensitivities, with empirical therapy initiated only after appropriate specimen collection.

### 2.7. Sample Size Calculation

Based on our institutional UTI rate of 47% and anticipating a 50% relative reduction with gentamicin irrigation, we calculated that 85 patients per group would provide 80% power to detect this difference at α = 0.05. Accounting for 15% dropout, we planned to enroll 200 patients total.

### 2.8. Statistical Analysis

The primary analysis followed intention-to-treat principles. However, given baseline imbalances despite randomization (standardized mean differences > 0.2 for key variables), we performed propensity score matching as a sensitivity analysis.

Propensity scores were calculated using logistic regression including: age, sex, body mass index, diabetes status, and ABO blood type. We performed 1:1 nearest-neighbor matching with a caliper of 0.10 standard deviations of the logit of the propensity score.

Categorical variables were compared using chi-square or Fisher's exact tests. Continuous variables were analyzed using *t*-tests or Mann–Whitney *U* tests as appropriate. Time-to-event analyses used Kaplan–Meier curves and log-rank tests. Multivariable logistic regression adjusted for residual imbalances after matching.

Subgroup analyses were prespecified for sex, donor type, and induction therapy. Statistical significance was set at *p* < 0.05 for the primary outcome. All analyses were performed using *R* version 4.2.0.

### 2.9. Ethical Considerations

The study was conducted according to Declaration of Helsinki principles. The institutional review board approved the protocol, and all participants provided written informed consent. An independent data safety monitoring board reviewed adverse events quarterly.

## 3. Results

Of the 212 eligible kidney transplant recipients, 65 (30.7%) were excluded prior to enrollment. Exclusion reasons included urethral stricture, active lower limb infection, perioperative complications, and ongoing antibiotic therapy (*n* = 1, 0.5% each) and logistical issues including patient withdrawal or noncompliance 61 (28.8%). The remaining 147 (69.3%) patients were randomized to receive either intraoperative bladder irrigation with gentamicin solution 64 (43.5%) or saline control 83 (56.5%)

### 3.1. Baseline Characteristics Before Matching

The baseline characteristics of the unmatched cohort are presented in Table 1. The control group was slightly younger (51.6 ± 14.2 vs 54.8 ± 13.7 years, SMD = 0.233) and had a lower proportion of male recipients (65.1% vs 73.4%, SMD = 0.182). The prevalence of diabetes mellitus was comparable between groups (18.1% vs 23.4%, SMD = 0.133), as were body mass index values (26.7 ± 5.1 vs 26.7 ± 4.5 kg/m^2^, SMD = 0.010). The distribution of ABO blood types was similar between groups (*p*=0.896).

### 3.2. Propensity Score Matching

Propensity score matching (1:1, caliper 0.10) using age, sex, BMI, diabetes, and ABO blood type yielded 49 matched pairs (*n* = 98). All baseline variables achieved good balance (SMD < 0.25), with matched cohorts similar in age (53.4 ± 13.8 vs 54.2 ± 14.1 years), male proportion (71.4% vs 73.5%), diabetes prevalence (22.4% both groups), and BMI (26.0 ± 4.9 vs 27.0 ± 4.4 kg/m^2^) (Table 2).

### 3.3. Transplant Characteristics

Table 3 presents the transplant-related characteristics of the matched cohort. The majority of patients received kidneys from living donors (65.3% control vs 69.4% gentamicin, *p*=0.829). Retransplantation rates were identical between groups (8.2%). Recipients had been on dialysis for a median of approximately 21 months, with no significant difference between groups (22.2 ± 20.1 vs 20.3 ± 17.5 months, *p*=0.619).

Notably, predialysis urine output showed a trend toward higher values in the control group (844.9 ± 548.1 vs 653.1 ± 494.6 mL, *p*=0.072, SMD = 0.367), though this did not reach statistical significance. Post-transplant parameters were similar, including length of stay (11.8 ± 5.2 vs 10.7 ± 5.3 days, *p*=0.289) and serum creatinine at discharge (1.77 ± 1.14 vs 1.56 ± 1.02 mg/dL, *p*=0.326).

### 3.4. Primary Outcome

The primary outcome of any UTI within 30 days post-transplant occurred in 13 patients (26.5%) in the control group compared to 8 patients (16.3%) in the gentamicin group (Table 4). This represented an absolute risk reduction of 10.2% (95% CI: −5.0%–25.4%) and a relative risk reduction of 38.5%. However, this difference did not reach statistical significance (*p*=0.325).

**Figure 1:**
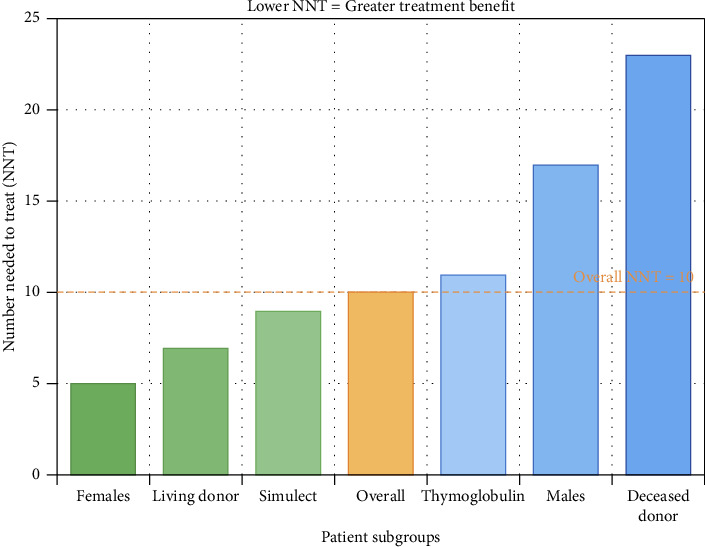
Number needed to treat shown in detail.

**Figure 2:**
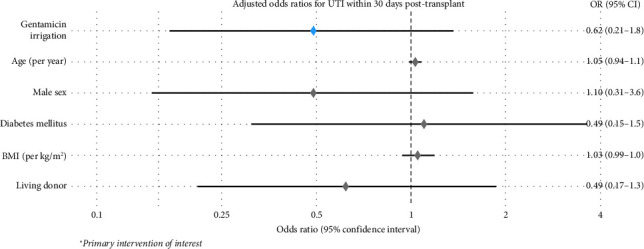
Adjusted odds ratio for UTI within 30 days post-transplant. Multivariant analysis forest plot.

Figure 1 shows in detail the numbers needed to treat in function of the subgroups; The number needed to treat (NNT) was 10, indicating that treating 10 patients with gentamicin bladder irrigation would prevent one UTI within the first 30 days post-transplant. In multivariable logistic regression analysis adjusting for age, sex, BMI, diabetes, and living donor status, the odds ratio for gentamicin versus control was 0.49 (95% CI: 0.17-1.36, *p*=0.178).  Figure 2 illustrates the odds ratios of the multivariant regression by a forest plot (Table 5).  Table 6 shows the efficacy measures for primary outcome.

### 3.5. Secondary Outcomes

The timing of UTI episodes revealed that most infections occurred early, with first episodes accounting for all primary outcome events. Recurrent UTIs were uncommon, with only 5 patients experiencing a second episode (6.1% control vs 4.1% gentamicin, *p*=1.000) and 1 patient in the control group experiencing a third episode.

**Figure 3:**
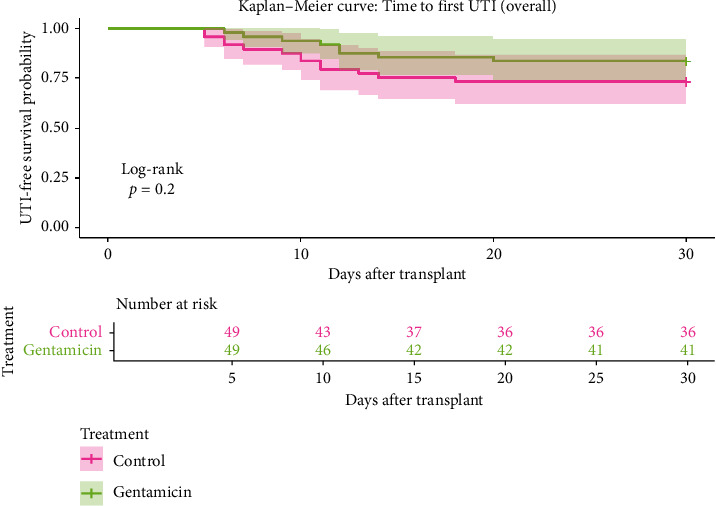
UTI-free survival probability.

Other infectious complications were infrequent and similarly distributed between groups. Bacteremia occurred in 4.1% of control patients versus 6.1% of gentamicin patients (*p*=1.000). Surgical site infections were observed in 6.1% versus 10.2%, respectively (*p*=0.712). One death occurred in the control group (2.0%) compared to none in the gentamicin group (*p*=1.000). UTI-free survival is shown in Figure 3.

Detailed mono-microbial infection is shown in supporting Tables S1–S3.

### 3.6. Subgroup Analyses (Figure 4, Table Supporting S5)

#### 3.6.1. Analysis by Sex

Subgroup analysis revealed striking differences in UTI incidence by sex (Table 7). Female recipients had a significantly higher overall UTI rate compared to males (40.7% vs 14.1%, *p*=0.004). This represented a nearly threefold increased risk among females.

The treatment effect of gentamicin varied substantially by sex. Among females, the UTI rate decreased from 50.0% (7/14) in the control group to 30.8% (4/13) in the gentamicin group, yielding an absolute risk reduction of 19.2% and an NNT of 5. In contrast, males showed a more modest reduction from 17.1% (6/35) to 11.1% (4/36), with an absolute risk reduction of 6.0% and an NNT of 17.

#### 3.6.2. Analysis by Donor Type

When stratified by donor type (Table 8), deceased donor recipients had significantly longer pretransplant dialysis duration (35.0 ± 18.1 vs 14.6 ± 15.5 months, *p* < 0.001) but paradoxically lower UTI rates (15.6% vs 24.2%, *p*=0.329). The treatment effect was more pronounced in living donor recipients (NNT = 7) compared to deceased donor recipients (NNT = 23).

#### 3.6.3. Analysis by Induction Therapy

Recipients of Simulect versus Thymoglobulin showed notable differences in baseline characteristics (Table 9), with Simulect recipients more likely to receive living donor kidneys (82.4% vs 51.1%, *p*=0.001) and having shorter dialysis duration (15.0 ± 15.1 vs 28.4 ± 20.1 months, *p* < 0.001). However, UTI rates were nearly identical between induction groups (21.6% vs 21.3%, *p*= 0.972), and the treatment effect of gentamicin was consistent regardless of induction therapy used (NNT of 9 for Simulect vs 11 for Thymoglobulin) (Supplement Table S4)

## 4. Discussion

**Scheme 1:**
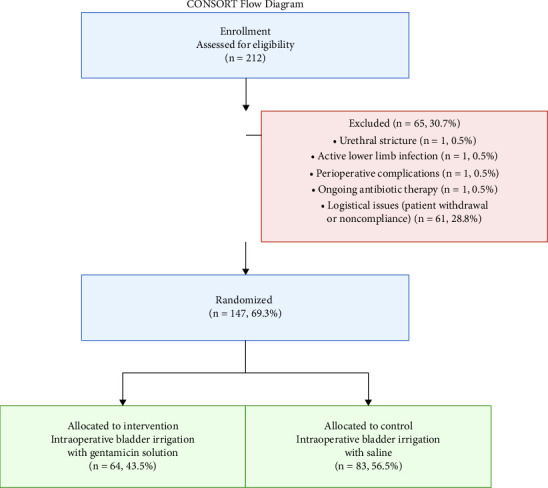
Consort flow diagram.

This randomized controlled trial evaluated intraoperative gentamicin bladder irrigation as a preventive strategy for early post-transplant UTIs. Our findings demonstrate a clinically meaningful 38.5% relative reduction in UTI incidence, with significant sex-specific differences that have important implications for personalized prevention strategies in kidney transplantation (Scheme 1).

The overall UTI incidence of 26.5% in our control group aligns with contemporary reports, confirming that despite advances in transplant care, UTIs remain a substantial burden [1–7]. The reduction to 16.3% with gentamicin irrigation, while not reaching statistical significance (*p* = 0.325), represents a clinically important effect size with a number needed to treat of 10. This compares favorably with systemic antibiotic prophylaxis, where Green et al.'s meta-analysis reported NNTs ranging from 7 to 17 for preventing bacteriuria [24]. Our results build upon the pioneering work of Salehipour et al., who reported significant UTI reduction using perioperative amikacin bladder irrigation in 100 kidney transplant recipients [21]. However, our study advances this concept through rigorous double-blinding, gentamicin utilization, and the revelation of crucial subgroup effects. The lack of statistical significance in our overall analysis likely reflects inadequate power rather than absence of effect, as post hoc analysis revealed only 30% power to detect the observed difference.

The most compelling finding was the marked sex-dependent treatment effect. Female recipients demonstrated both higher baseline risk (40.7% vs 14.1%, *p*=0.004) and greater treatment benefit (NNT 5 vs 17). This sex disparity in UTI susceptibility is well established in both general and transplant populations [15–17], yet the differential treatment response is novel and clinically important. The enhanced efficacy in females likely reflects anatomical and microbiological factors. The shorter female urethra facilitates bacterial ascension, creating a higher baseline bacterial load that may be more amenable to local antimicrobial intervention. Additionally, the female urogenital microbiome differs substantially from males, potentially influencing antimicrobial efficacy [25]. These findings suggest that universal prevention strategies may be suboptimal and that sex-specific protocols warrant consideration.

The efficacy of intraoperative bladder irrigation likely stems from achieving high local antimicrobial concentrations during a critical window of vulnerability. Surgical manipulation, catheter insertion, and ureteral stenting create opportunities for bacterial colonization. By administering gentamicin during bladder filling for ureteroneocystostomy, we maximize contact time when the urothelium is most susceptible to colonization. Gentamicin's pharmacokinetic properties make it ideal for topical use—its poor systemic absorption from intact bladder mucosa minimizes toxicity while maintaining bactericidal concentrations at the urothelial surface; we measured blood concentration both before and after irrigation and received undetectable blood concentration of gentamicin [26]. The drug's concentration-dependent killing and post-antibiotic effect provide sustained antimicrobial activity beyond the irrigation period [27]. This contrasts with previous studies using cephalotin, which showed no benefit, possibly due to its time-dependent killing kinetics being less suited for single-dose topical application [28].

Our pathogen distribution mirrors global trends, with *Klebsiella pneumoniae* and *Escherichia coli* predominating. However, the high proportion of *Klebsiella* (46.2% of control UTIs) exceeds most reports and may reflect local epidemiology or the high-risk nature of our population [18, 19]. Notably, gentamicin irrigation reduced *Klebsiella* UTIs by 33%, suggesting good activity against local strains. The emergence of multidrug-resistant organisms in transplant recipients remains concerning [12–14]. While we observed no increased resistance with gentamicin use, longer follow-up is needed. The theoretical advantage of topical administration is reduced selective pressure compared to systemic prophylaxis, though this requires confirmation through resistance surveillance.

The protective effect of gentamicin bladder irrigation was observed across all analyzed subgroups, with odds ratios consistently favoring the intervention. However, the magnitude of benefit varied considerably, with the greatest effect observed in high-risk populations, particularly female recipients and those receiving living donor kidneys. Living donor recipients showed greater benefit (NNT 7) than deceased donor recipients (NNT 23). This paradoxical finding, given that deceased donor recipients typically have higher infection risk, may reflect the confounding effect of dialysis duration. Deceased donor recipients had significantly longer pretransplant dialysis (35.0 vs 14.6 months, *p* < 0.001), which associates with altered immunity and potentially different baseline flora [29]. The consistent efficacy across induction regimens (NNT 9 for basiliximab vs 11 for antithymocyte globulin) suggests the intervention works independently of immunosuppression intensity, supporting broad applicability despite trends toward more potent induction protocols [30].

The excellent safety profile, with no increase in adverse events, supports clinical applicability. Unlike systemic aminoglycosides, topical administration avoids nephrotoxicity and ototoxicity risks [31]. Similar bacteremia rates between groups (4.1% vs 6.1%) indicate no increased risk of systemic infection, addressing theoretical concerns about disrupting protective bladder flora. Implementation requires minimal resources beyond standard surgical supplies, and the intervention integrates seamlessly into existing surgical workflow, as bladder filling is routinely performed for ureteroneocystostomy. This contrasts with postoperative irrigation protocols requiring additional procedures and monitoring [32].

Our study's methodological rigor includes randomization, double-blinding, comprehensive outcome assessment, and detailed subgroup analyses. The use of propensity score matching to address baseline imbalances strengthens causal inference while acknowledging randomization imperfections. Intensive microbiological surveillance captured both symptomatic and asymptomatic infections, providing complete outcome ascertainment.

However, several limitations warrant consideration. The single-center design may limit generalizability, though our patient characteristics and outcomes align with international data. The study was underpowered for the primary endpoint, achieving only 30% power to detect the observed difference, reflecting optimistic effect size assumptions during planning. We cannot assess long-term outcomes including late UTIs, resistance development, or graft survival impact. Several important clinical variables were not analyzed: the impact of ureteral stent duration on UTI incidence and whether earlier stent removal might enhance or diminish the protective effect of gentamicin irrigation; the influence of maintenance immunosuppression parameters including mycophenolate doses, steroid tapering schedules, and calcineurin inhibitor trough levels on infection risk; and potential interactions between systemic antimicrobial prophylaxis and topical gentamicin irrigation. Our primary analysis did not stratify between symptomatic and asymptomatic infections, though post hoc review showed similar proportions in both groups. Additionally, the unexpected similarity in UTI rates between basiliximab and antithymocyte globulin induction (21.6% vs 21.3%) deserves further investigation, as this contradicts the anticipated higher infection risk with more potent immunosuppression. Most critically, we acknowledge that technical difficulties preventing trial registration may raise concerns about transparency and could potentially limit the robustness of our data interpretation. This limitation, while not affecting study conduct or protocol adherence, represents a deviation from optimal clinical trial standards that we recognize may influence the perceived validity of our findings.

Our findings support a paradigm shift from universal to risk-stratified UTI prevention. Female recipients, with an NNT of 5, should be prioritized for gentamicin irrigation. For male recipients with lower baseline risk and modest benefit (NNT 17), decisions could be individualized based on additional risk factors. Cost-effectiveness likely favors the intervention, particularly in high-risk groups—preventing one UTI in every 5–10 treated patients would offset drug costs through reduced hospitalization, diagnostic testing, and treatment expenses. Additionally, preventing early infections may have unmeasured benefits on graft function and patient quality of life [33].

These results provide strong rationale for a definitive multicenter trial. Based on our effect sizes, 300 patients would provide adequate power for the overall population, while 140 females would suffice for this high-risk subgroup. Sex-stratified randomization should be employed to ensure adequate female representation. Several research questions warrant investigation: optimal gentamicin concentration and volume, efficacy in deceased donor and pediatric recipients, long-term effects on resistance patterns, combination with other preventive strategies, and cost-effectiveness modeling including quality-adjusted life years. Additionally, investigating urinary and vaginal microbiome changes could elucidate mechanisms underlying sex-specific effects and identify biomarkers for treatment response [34].

**Figure 5:**
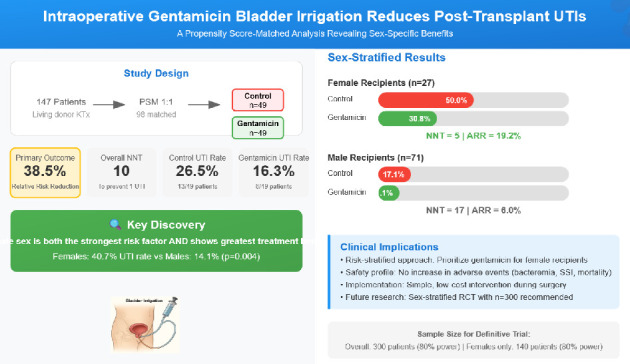
Central image.

Intraoperative gentamicin bladder irrigation represents a promising strategy for preventing early post-transplant UTIs, with particular efficacy in female recipients. While our study was underpowered for the primary endpoint, the magnitude of effect, excellent safety profile, and striking subgroup differences provide compelling evidence for selective implementation. Centers might consider adopting this low-risk intervention for high-risk recipients while awaiting confirmatory trials. Given the persistent burden of post-transplant UTIs and limitations of current strategies, continued innovation in prevention approaches remains critical for optimizing transplant outcomes (Figure 5).

## Figures and Tables

**Figure 4:**
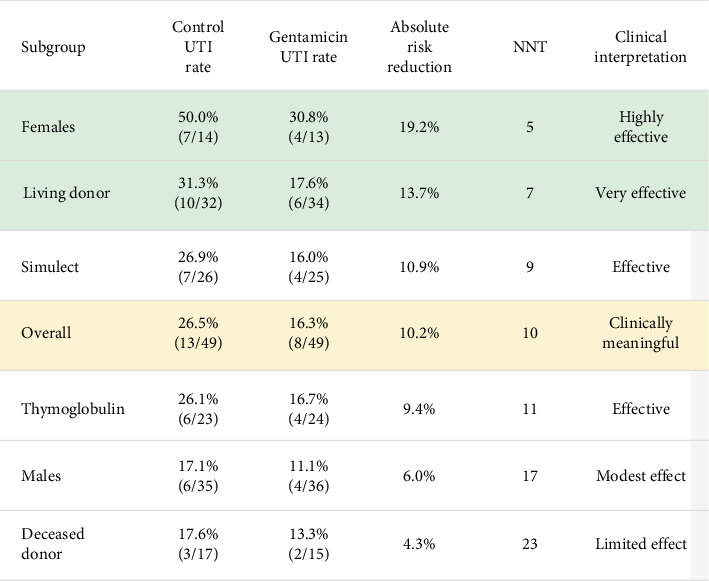
Detailed subgroup analysis.

**Table 1:** Baseline characteristics before propensity score matching.

Characteristic	Control (*n* = 83)	Gentamicin (*n* = 64)	*p* value	SMD
Age, years	51.6 ± 14.2	54.8 ± 13.7	0.165	0.233
Male sex, *n* (%)	54 (65.1)	47 (73.4)	0.365	0.182
Body mass index, kg/m^2^	26.7 ± 5.1	26.7 ± 4.5	0.952	0.010
Diabetes mellitus, *n* (%)	15 (18.1)	15 (23.4)	0.553	0.133
ABO blood type, *n* (%)			0.896	0.129
-Type A	31 (37.3)	22 (34.4)		
-Type AB	5 (6.0)	3 (4.7)		
-Type B	17 (20.5)	12 (18.8)		
-Type O	30 (36.1)	27 (42.2)		

*Note:* Data presented as mean ± SD or *n* (%).

Abbreviation: SMD = standardized mean difference.

**Table 2:** Baseline characteristics after propensity score matching.

Characteristic	Control (*n* = 49)	Gentamicin (*n* = 49)	*p* value	SMD
Age, years	53.4 ± 13.8	54.2 ± 14.1	0.778	0.057
Male sex, *n* (%)	35 (71.4)	36 (73.5)	1.000	0.046
Body mass index, kg/m^2^	26.0 ± 4.9	27.0 ± 4.4	0.276	0.221
Diabetes mellitus, *n* (%)	11 (22.4)	11 (22.4)	1.000	< 0.001
ABO blood type, *n* (%)			0.994	0.058
-Type A	17 (34.7)	17 (34.7)		
-Type AB	2 (4.1)	2 (4.1)		
-Type B	9 (18.4)	8 (16.3)		
-Type O	21 (42.9)	22 (44.9)		

*Note:* Data presented as mean ± SD or *n* (%), values < 0.25 indicate good balance.

Abbreviation: SMD = standardized mean difference.

**Table 3:** Transplant characteristics and perioperative variables (matched cohort).

Characteristic	Control (*n* = 49)	Gentamicin (*n* = 49)	*p* value
*Donor characteristics*			
Living donor, *n* (%)	32 (65.3)	34 (69.4)	0.829
DCD donor, *n* (%)	1 (2.0)	0 (0.0)	1.000

*Recipient characteristics*			
Retransplant, *n* (%)	4 (8.2)	4 (8.2)	1.000
Dialysis duration, months	22.2 ± 20.1	20.3 ± 17.5	0.619
Predialysis urine output, mL	844.9 ± 548.1	653.1 ± 494.6	0.072

*Post-transplant parameters*			
Length of stay, days	11.8 ± 5.2	10.7 ± 5.3	0.289
Creatinine at discharge, mg/dL	1.77 ± 1.14	1.56 ± 1.02	0.326
Last creatinine, mg/dL	1.59 ± 0.95	1.44 ± 0.61	0.343

*Note:* Data presented as mean ± SD or *n* (%).

Abbreviations: DCD = donation after cardiac death, SMD = standardized mean difference.

**Table 4:** Clinical outcomes (matched cohort).

Outcome	Control (*n* = 49)	Gentamicin (*n* = 49)	Risk difference (95% CI)	*p* value
*Primary outcome*				
Any UTI within 30 days, *n* (%)	13 (26.5)	8 (16.3)	−10.2 (−25.4 to 5.0)	0.325

**Secondary outcomes**				
**UTI timing**				

-First UTI episode, *n* (%)	13 (26.5)	8 (16.3)	−10.2 (−25.4 to 5.0)	0.325
-Second UTI episode, *n* (%)	3 (6.1)	2 (4.1)	−2.0 (−9.9 to 5.8)	1.000
-Third UTI episode, *n* (%)	1 (2.0)	0 (0.0)	−2.0 (−7.6 to 3.5)	1.000

*Other infections*				
-Bacteremia, *n* (%)	2 (4.1)	3 (6.1)	2.0 (−6.7–10.8)	1.000
-Surgical site infection, *n* (%)	3 (6.1)	5 (10.2)	4.1 (−6.8–15.0)	0.712

*Mortality*				
-Death, *n* (%)	1 (2.0)	0 (0.0)	−2.0 (−7.6 to 3.5)	1.000

*Note:* Data presented as *n* (%).

Abreviations: CI = confidence interval, UTI = urinary tract infection.

**Table 5:** Multivariable logistic regression analysis for any UTI.

Variable	Odds ratio	95% CI	*p* value
Gentamicin irrigation	0.49	0.17–1.36	0.178
Age (per year)	1.03	0.99–1.08	0.162
Male sex	0.49	0.15–1.58	0.227
Diabetes mellitus	1.10	0.31–3.63	0.872
BMI (per kg/m^2^)	1.05	0.94–1.19	0.381
Living donor	0.62	0.21–1.87	0.388

Abbreviations: BMI = body mass index, CI = confidence interval.

**Table 6:** Efficacy measures for primary outcome.

Measure	Value (95% CI)
Control group UTI rate	26.5%
Gentamicin group UTI rate	16.3%
Absolute risk reduction	10.2% (−5.0–25.4)
Relative risk	0.62 (0.28–1.35)
Relative risk reduction	38.5%
Number needed to treat	10 (4 to ∞)
Odds ratio (adjusted)^∗^	0.49 (0.17–1.36)

^∗^Adjusted for age, sex, BMI, diabetes mellitus, and living donor status.

**Table 7:** Baseline characteristics by sex (matched cohort).

Characteristic	Females (*n* = 27)	Males (*n* = 71)	*p* value
Treatment group, *n* (%)			0.919
-Control	14 (51.9)	35 (49.3)	
-Gentamicin	13 (48.1)	36 (50.7)	
Age, years	51.4 ± 14.5	54.6 ± 13.6	0.309
Body mass index, kg/m^2^	26.3 ± 4.8	26.6 ± 4.6	0.784
Diabetes mellitus, *n* (%)	5 (18.5)	17 (23.9)	0.564
Living donor, *n* (%)	17 (63.0)	49 (69.0)	0.564
Dialysis duration, months	23.1 ± 19.8	20.6 ± 18.6	0.568

*Clinical Outcomes, n (%)*			
Any UTI	11 (40.7)	10 (14.1)	0.004
-UTI episode 1	11 (40.7)	10 (14.1)	0.004
-UTI episode 2	3 (11.1)	2 (2.8)	0.096
-UTI episode 3	0 (0.0)	1 (1.4)	0.537
Bacteremia	1 (3.7)	4 (5.6)	0.702
Surgical site infection	3 (11.1)	5 (7.0)	0.509
Death	0 (0.0)	1 (1.4)	0.537

*Note:* Data presented as mean ± SD or *n* (%).

**Table 8:** Baseline characteristics by donor type (matched cohort).

Characteristic	Deceased donor (*n* = 32)	Living donor (*n* = 66)	*p* value
Treatment group, *n* (%)			0.829
-Control	17 (53.1)	32 (48.5)	
-Gentamicin	15 (46.9)	34 (51.5)	
Age, years	55.7 ± 11.5	52.7 ± 14.8	0.318
Male sex, *n* (%)	22 (68.8)	49 (74.2)	0.564
Body mass index, kg/m^2^	26.9 ± 5.1	26.3 ± 4.4	0.554
Diabetes mellitus, *n* (%)	7 (21.9)	15 (22.7)	0.924
Dialysis duration, months	35.0 ± 18.1	14.6 ± 15.5	< 0.001

*Clinical Outcomes, n (%)*			
Any UTI	5 (15.6)	16 (24.2)	0.329
-UTI episode 1	5 (15.6)	16 (24.2)	0.329
-UTI episode 2	1 (3.1)	4 (6.1)	0.533
-UTI episode 3	0 (0.0)	1 (1.5)	0.483
Bacteremia	1 (3.1)	4 (6.1)	0.533
Surgical site infection	3 (9.4)	5 (7.6)	0.759
Death	0 (0.0)	1 (1.5)	0.483

*Note:* Data presented as mean ± SD or *n* (%).

**Table 9:** Baseline characteristics by induction therapy (matched cohort).

Characteristic	Simulect (*n* = 51)	Thymoglobulin (*n* = 47)	*p* value
Treatment group, *n* (%)			0.852
-Control	26 (51.0)	23 (48.9)	
-Gentamicin	25 (49.0)	24 (51.1)	
Age, years	51.5 ± 14.8	56.3 ± 12.3	0.084
Male sex, *n* (%)	38 (74.5)	33 (70.2)	0.635
Body mass index, kg/m^2^	26.5 ± 4.9	26.5 ± 4.3	0.968
Diabetes mellitus, *n* (%)	10 (19.6)	12 (25.5)	0.480
Living donor, *n* (%)	42 (82.4)	24 (51.1)	0.001
Dialysis duration, months	15.0 ± 15.1	28.4 ± 20.1	< 0.001

*Clinical Outcomes, n (%)*			
Any UTI	11 (21.6)	10 (21.3)	0.972
-UTI episode 1	11 (21.6)	10 (21.3)	0.972
-UTI episode 2	2 (3.9)	3 (6.4)	0.576
-UTI episode 3	0 (0.0)	1 (2.1)	0.299
Bacteremia	1 (2.0)	4 (8.5)	0.141
Surgical site infection	2 (3.9)	6 (12.8)	0.111
Death	0 (0.0)	1 (2.1)	0.299

*Note:* Data presented as mean ± SD or *n* (%).

## Data Availability

The datasets used and analyzed during the current study are available from the corresponding author upon reasonable request.

## Nomenclature

ARR: Absolute risk reduction
AST: American Society of Transplantation
BMI: Body mass index
CFU: Colony-forming units
CI: Confidence interval
CONSORT: Consolidated Standards of Reporting Trials
DCD: Donation after cardiac death
IRB: Institutional Review Board
KDIGO: Kidney Disease: Improving Global Outcomes
NNT: Number needed to treat
OR: Odds ratio
PSM: Propensity score matching
RCT: Randomized controlled trial
SMD: Standardized mean difference
UTI: Urinary tract infection

## Supporting Information

Supplemental Table S1. UTI-1 Microbiology Summary Comprehensive microbiological analysis of urinary isolates from the UTI-1 cohort, comparing organism distribution between control and gentamicin groups. Presents total isolates, clinical UTI cases, and antibiotic-treated cases with corresponding percentages for 18 different pathogens.

Supplemental Table S2. UTI-2 Microbiology Summary Microbiological profile of urinary isolates from the UTI-2 cohort, detailing organism distribution across treatment groups. Includes 12 pathogen types with their prevalence in clinical UTIs and antibiotic treatment rates.

Supplemental Table S3. UTI-3 Microbiology Summary Microbiological findings from the UTI-3 cohort, presenting distribution of 10 urinary pathogens between control and gentamicin groups, with clinical UTI incidence and treatment percentages.

Supplemental Table S4. Subgroup Analysis of Any UTI by Clinical Characteristics (Matched Cohort) This table presents the distribution of urinary tract infections across different clinical subgroups in the matched cohort (*n* = 98). UTI rates are compared between treatment groups (gentamicin vs control) and across patient characteristics including sex, donor type, and induction therapy. The analysis reveals significant differences in baseline UTI risk by sex (*p* = 0.004), with females demonstrating nearly threefold higher infection rates compared to males. No significant differences were observed between donor types or induction regimens. UTI = urinary tract infection. ∗*P* values derived from Fisher's exact test for comparisons with expected cell counts < 5, and chi-square test otherwise.

Supplemental Table S5. Treatment Effect by Subgroup (Any UTI Outcome) This table demonstrates the differential treatment effect of gentamicin bladder irrigation across predefined subgroups. The absolute risk reduction and number needed to treat (NNT) vary substantially by patient characteristics. Female recipients show the greatest benefit with an NNT of 5, while males require treating 17 patients to prevent one UTI. Living donor recipients (NNT = 7) respond better than deceased donor recipients (NNT = 23). The treatment effect remains consistent across induction therapies. These findings support a risk-stratified approach to UTI prevention, prioritizing intervention in high-risk subgroups. NNT = Number needed to treat; calculated as 1/absolute risk reduction. ∗*p* values from Fisher's exact test or chi-square test as appropriate.
