# Supplementary material for: Evaluation of Intraoperative Gentamicin Bladder Irrigation for Mitigation of Urinary Tract Infections After Kidney Transplantation: A Propensity Score–Matched Analysis of a Randomized Controlled Trial
Source: J Transplant. 2025 Nov 29;2025:9377493. doi: 10.1155/joot/9377493 (PMC12681396; doi:10.1155/joot/9377493)
Supplement: Supporting Information — Additional supporting information can be found online in the Supporting Information section. [file 9377493.f1.docx]

Supplements

## Supplemental table S1. UTI-1 Microbiology Summary

| Organism | Total_Control | Total_Genta | UTI_Control | UTI_Genta | ABX_Control | ABX_Genta | % Clinical UTI (Control) | % Clinical UTI (Genta) | % Treated (Control) | % Treated (Genta) |
| --- | --- | --- | --- | --- | --- | --- | --- | --- | --- | --- |
| Kleb. pneumoniae | 13.0 | 6.0 | 6.0 | 4.0 | 6.0 | 4.0 | 46.2 | 66.7 | 46.2 | 66.7 |
| Pseud. aeruginosa | 6.0 | 3.0 | 4.0 | 1.0 | 4.0 | 1.0 | 66.7 | 33.3 | 66.7 | 33.3 |
| Staph. epidermidis | 6.0 | 3.0 | 1.0 | 0.0 | 1.0 | 0.0 | 16.7 | 0.0 | 16.7 | 0.0 |
| Escherichia coli | 5.0 | 7.0 | 1.0 | 2.0 | 1.0 | 2.0 | 20.0 | 28.6 | 20.0 | 28.6 |
| Enteroc. faecalis | 5.0 | 0.0 | 2.0 | 0.0 | 2.0 | 0.0 | 40.0 | nan | 40.0 | nan |
| Enterococcus spp. | 4.0 | 3.0 | 3.0 | 0.0 | 3.0 | 0.0 | 75.0 | 0.0 | 75.0 | 0.0 |
| Staph. haemolyticus | 3.0 | 1.0 | 0.0 | 0.0 | 0.0 | 0.0 | 0.0 | 0.0 | 0.0 | 0.0 |
| Morganella morganii | 3.0 | 0.0 | 0.0 | 0.0 | 0.0 | 0.0 | 0.0 | nan | 0.0 | nan |
| Enterococcus faecium | 2.0 | 1.0 | 0.0 | 0.0 | 0.0 | 0.0 | 0.0 | 0.0 | 0.0 | 0.0 |
| Yeasts | 2.0 | 0.0 | 1.0 | 0.0 | 1.0 | 0.0 | 50.0 | nan | 50.0 | nan |
| Enterob cloacae comp | 1.0 | 2.0 | 1.0 | 1.0 | 1.0 | 1.0 | 100.0 | 50.0 | 100.0 | 50.0 |
| Candida glabrata | 1.0 | 0.0 | 0.0 | 0.0 | 0.0 | 0.0 | 0.0 | nan | 0.0 | nan |
| Citrobacter freundii | 1.0 | 0.0 | 0.0 | 0.0 | 0.0 | 0.0 | 0.0 | nan | 0.0 | nan |
| Serratia marcescens | 1.0 | 0.0 | 0.0 | 0.0 | 0.0 | 0.0 | 0.0 | nan | 0.0 | nan |
| Citrobacter koseri | 0.0 | 1.0 | 0.0 | 1.0 | 0.0 | 1.0 | nan | 100.0 | nan | 100.0 |
| Klebsiella aerogenes | 0.0 | 1.0 | 0.0 | 0.0 | 0.0 | 0.0 | nan | 0.0 | nan | 0.0 |
| Klebsiella oxytoca | 0.0 | 1.0 | 0.0 | 0.0 | 0.0 | 0.0 | nan | 0.0 | nan | 0.0 |
| Proteus mirabilis | 0.0 | 1.0 | 0.0 | 1.0 | 0.0 | 1.0 | nan | 100.0 | nan | 100.0 |

## Supplemental table S2.UTI-2 Microbiology Summary

| Organism | Total_Control | Total_Genta | UTI_Control | UTI_Genta | ABX_Control | ABX_Genta | % Clinical UTI (Control) | % Clinical UTI (Genta) | % Treated (Control) | % Treated (Genta) |
| --- | --- | --- | --- | --- | --- | --- | --- | --- | --- | --- |
| Kleb. pneumoniae | 12.0 | 2.0 | 2.0 | 0.0 | 3.0 | 0.0 | 16.7 | 0.0 | 25.0 | 0.0 |
| Escherichia coli | 6.0 | 8.0 | 2.0 | 1.0 | 2.0 | 1.0 | 33.3 | 12.5 | 33.3 | 12.5 |
| Enterococcus spp. | 3.0 | 2.0 | 0.0 | 0.0 | 0.0 | 0.0 | 0.0 | 0.0 | 0.0 | 0.0 |
| Yeasts | 3.0 | 2.0 | 1.0 | 0.0 | 1.0 | 0.0 | 33.3 | 0.0 | 33.3 | 0.0 |
| Enterococcus faecium | 2.0 | 0.0 | 0.0 | 0.0 | 0.0 | 0.0 | 0.0 | nan | 0.0 | nan |
| Staph. epidermidis | 2.0 | 0.0 | 0.0 | 0.0 | 0.0 | 0.0 | 0.0 | nan | 0.0 | nan |
| Pseud. aeruginosa | 1.0 | 1.0 | 0.0 | 0.0 | 0.0 | 1.0 | 0.0 | 0.0 | 0.0 | 100.0 |
| Enteroc. faecalis | 1.0 | 0.0 | 0.0 | 0.0 | 0.0 | 0.0 | 0.0 | nan | 0.0 | nan |
| Proteus mirabilis | 1.0 | 0.0 | 0.0 | 0.0 | 0.0 | 0.0 | 0.0 | nan | 0.0 | nan |
| Staph. haemolyticus | 1.0 | 0.0 | 0.0 | 0.0 | 0.0 | 0.0 | 0.0 | nan | 0.0 | nan |
| Enterob cloacae comp | 0.0 | 2.0 | 0.0 | 1.0 | 0.0 | 1.0 | nan | 50.0 | nan | 50.0 |
| Citrobacter koseri | 0.0 | 1.0 | 0.0 | 1.0 | 0.0 | 1.0 | nan | 100.0 | nan | 100.0 |

## Supplemental table S3. UTI-3 Microbiology Summary

| Organism | Total_Control | Total_Genta | UTI_Control | UTI_Genta | ABX_Control | ABX_Genta | % Clinical UTI (Control) | % Clinical UTI (Genta) | % Treated (Control) | % Treated (Genta) |
| --- | --- | --- | --- | --- | --- | --- | --- | --- | --- | --- |
| Kleb. pneumoniae | 5.0 | 1.0 | 1.0 | 0.0 | 1.0 | 0.0 | 20.0 | 0.0 | 20.0 | 0.0 |
| Escherichia coli | 4.0 | 3.0 | 0.0 | 0.0 | 1.0 | 0.0 | 0.0 | 0.0 | 25.0 | 0.0 |
| Enterococcus spp. | 3.0 | 1.0 | 0.0 | 0.0 | 0.0 | 0.0 | 0.0 | 0.0 | 0.0 | 0.0 |
| Enteroc. faecalis | 2.0 | 0.0 | 0.0 | 0.0 | 0.0 | 0.0 | 0.0 | nan | 0.0 | nan |
| Staph. haemolyticus | 2.0 | 0.0 | 0.0 | 0.0 | 0.0 | 0.0 | 0.0 | nan | 0.0 | nan |
| Yeasts | 1.0 | 1.0 | 1.0 | 0.0 | 1.0 | 0.0 | 100.0 | 0.0 | 100.0 | 0.0 |
| Enterococcus faecium | 1.0 | 0.0 | 0.0 | 0.0 | 0.0 | 0.0 | 0.0 | nan | 0.0 | nan |
| Lactobacillus sp. | 1.0 | 0.0 | 0.0 | 0.0 | 0.0 | 0.0 | 0.0 | nan | 0.0 | nan |
| Enterob cloacae comp | 0.0 | 1.0 | 0.0 | 0.0 | 0.0 | 0.0 | nan | 0.0 | nan | 0.0 |
| Pseud. aeruginosa | 0.0 | 1.0 | 0.0 | 0.0 | 0.0 | 1.0 | nan | 0.0 | nan | 100.0 |

table Supplement S4**. Treatment Effect by Subgroup (Any UTI Outcome)**

| **Subgroup** | **Control UTI Rate** | **Gentamicin UTI Rate** | **Absolute Risk Reduction** | **NNT** |
| --- | --- | --- | --- | --- |
| **Overall** | 26.5% (13/49) | 16.3% (8/49) | 10.2% | 10 |
| **Sex** |  |  |  |  |
| - Females | 50.0% (7/14) | 30.8% (4/13) | 19.2% | 5 |
| - Males | 17.1% (6/35) | 11.1% (4/36) | 6.0% | 17 |
| **Donor Type** |  |  |  |  |
| - Deceased | 17.6% (3/17) | 13.3% (2/15) | 4.3% | 23 |
| - Living | 31.3% (10/32) | 17.6% (6/34) | 13.7% | 7 |
| **Induction** |  |  |  |  |
| - Simulect | 26.9% (7/26) | 16.0% (4/25) | 10.9% | 9 |
| - Thymoglobulin | 26.1% (6/23) | 16.7% (4/24) | 9.4% | 11 |

NNT = Number needed to treat

**Table Supplement S5. Subgroup Analysis of Any UTI by Clinical Characteristics (Matched Cohort)**

| **Subgroup** | **Category** | **N** | **UTI Events** | **UTI Rate (%)** | **P value*** |
| --- | --- | --- | --- | --- | --- |
| **Treatment** |  |  |  |  |  |
|  | Control | 49 | 13 | 26.5 | 0.325 |
|  | Gentamicin | 49 | 8 | 16.3 |  |
| **Sex** |  |  |  |  |  |
|  | Female | 27 | 11 | 40.7 | 0.004 |
|  | Male | 71 | 10 | 14.1 |  |
| **Donor Type** |  |  |  |  |  |
|  | Deceased | 32 | 5 | 15.6 | 0.329 |
|  | Living | 66 | 16 | 24.2 |  |
| **Induction** |  |  |  |  |  |
|  | Simulect | 51 | 11 | 21.6 | 0.972 |
|  | Thymoglobulin | 47 | 10 | 21.3 |  |

**Supplementary Description**

**Supplemental Table S1. UTI-1 Microbiology Summary** Comprehensive microbiological analysis of urinary isolates from the UTI-1 cohort, comparing organism distribution between control and gentamicin groups. Presents total isolates, clinical UTI cases, and antibiotic-treated cases with corresponding percentages for 18 different pathogens.

**Supplemental Table S2. UTI-2 Microbiology Summary** Microbiological profile of urinary isolates from the UTI-2 cohort, detailing organism distribution across treatment groups. Includes 12 pathogen types with their prevalence in clinical UTIs and antibiotic treatment rates.

**Supplemental Table S3. UTI-3 Microbiology Summary** Microbiological findings from the UTI-3 cohort, presenting distribution of 10 urinary pathogens between control and gentamicin groups, with clinical UTI incidence and treatment percentages.

**Supplemental Table S4. Subgroup Analysis of Any UTI by Clinical Characteristics (Matched Cohort)** This table presents the distribution of urinary tract infections across different clinical subgroups in the matched cohort (n=98). UTI rates are compared between treatment groups (gentamicin vs control) and across patient characteristics including sex, donor type, and induction therapy. The analysis reveals significant differences in baseline UTI risk by sex (p=0.004), with females demonstrating nearly three-fold higher infection rates compared to males. No significant differences were observed between donor types or induction regimens. UTI = urinary tract infection. *P values derived from Fisher's exact test for comparisons with expected cell counts <5, and chi-square test otherwise.

**Supplemental Table S5. Treatment Effect by Subgroup (Any UTI Outcome)** This table demonstrates the differential treatment effect of gentamicin bladder irrigation across predefined subgroups. The absolute risk reduction and number needed to treat (NNT) vary substantially by patient characteristics. Female recipients show the greatest benefit with an NNT of 5, while males require treating 17 patients to prevent one UTI. Living donor recipients (NNT=7) respond better than deceased donor recipients (NNT=23). The treatment effect remains consistent across induction therapies. These findings support a risk-stratified approach to UTI prevention, prioritizing intervention in high-risk subgroups. NNT = Number needed to treat; calculated as 1/absolute risk reduction. *P values from Fisher's exact test or chi-square test as appropriate.
